# Serum lactate dehydrogenase isoenzyme 1 as a prognostic predictor for metastatic testicular germ cell tumours

**DOI:** 10.1054/bjoc.2000.1449

**Published:** 2000-11

**Authors:** F E von Eyben, E L Madsen, F Liu, R Amato, H Fritsche

**Affiliations:** Medical Research Unit, Amtsrådhuset, Torvet, PO Box 142, Ringkoebing, Denmark; Department of Oncology, Soenderborg Hospital, Denmark; Division of Clinical Chemistry, Department of Genitourinary Medical Oncology, Texas University MD Andersen Cancer Center, Honston, USA


**Table 1** Serum levels of studied hormones at 16 and 27 completed weeks of gestation among pregnant women in Boston, USA ($n$ = 304) and in Shanghai, China ($n$ = 334), stratified by maternal age

| Hormone | | Age ≤ 20 Sample 1 Mean | Age ≤ 20 Sample 2 Mean | Age 20–24 Sample 1 Mean | Age 20–24 Sample 2 Mean | Age 25–29 Sample 1 Mean | Age 25–29 Sample 2 Mean | Age 30+ Sample 1 Mean | Age 30+ Sample 2 Mean |
|---|---|---|---|---|---|---|---|---|---|
| Estradiol (E2) | Boston | 16.7 | 47.7 | 14.7 | 38.1 | 15.8 | 39.9 | 13.4 | 38.8 |
| nmol $l^{-1}$ | Shanghai | 17.0 | – | 20.7 | 49.5 | 20.3 | 46.9 | 21.9 | 44.8 |
| Estriol (E3) | Boston | 3.01 | 14.3 | 3.7 | 14.6 | 4.1 | 13.5 | 3.7 | 14.1 |
| nmol $l^{-1}$ | Shanghai | 5.0 | – | 6.1 | 22.6 | 6.4 | 20.8 | 6.7 | 21.2 |
| Prolactin | Boston | 25.9 | 80.1 | 39.2 | 65.2 | 47.2 | 89.6 | 44.0 | 92.4 |
| µg $l^{-1}$ | Shanghai | 30.0 | – | 58.5 | 111.5 | 67.0 | 123.5 | 71.7 | 119.7 |
| Progesterone | Boston | 88.6 | 167.0 | 104.9 | 248.3 | 136.3 | 263.5 | 132.6 | 263.9 |
| nmol $l^{-1}$ | Shanghai | 129.0 | – | 143.1 | 249.5 | 141.5 | 237.2 | 148.4 | 262.5 |
| Growth hormone | Boston | 13.7 | 1.3 | 1.4 | 0.6 | 3.3 | 0.9 | 2.9 | 0.9 |
| mU $l^{-1}$ | Shanghai | 5.9 | – | 4.2 | 1.9 | 3.3 | 1.5 | 2.8 | 1.7 |
| Albumin | Boston | 37.4 | 32.5 | 41.6 | 37.4 | 40.5 | 37.0 | 40.1 | 36.3 |
| g $l^{-1}$ | Shanghai | 44.7 | – | 43.6 | 40.0 | 42.8 | 39.1 | 42.3 | 38.9 |
| SHBG[a] | Boston | 417.3 | 344.2 | 312.6 | 417.1 | 381.1 | 431.8 | 356.1 | 423.7 |
| nmol $l^{-1}$ | Shanghai | 447.1 | – | 437.7 | 496.4 | 416.0 | 446.9 | 428.4 | 475.0 |

[a]Sex hormone-binding globulin.

**Table 2** Serum levels of studied hormones at 16 and 27 completed weeks of gestation among pregnant women in Boston, USA ($n$ = 304) and in Shanghai, China ($n$ = 334), stratified by maternal parity

| Hormone | | No previous livebirths Sample 1 Mean | No previous livebirths Sample 2 Mean | One previous livebirth Sample 1 Mean | One previous livebirth Sample 2 Mean |
|---|---|---|---|---|---|
| Estradiol (E2) | Boston | 15.4 | 42.2 | 11.8 | 33.9 |
| nmol $l^{-1}$ | Shanghai | 20.8 | 48.4 | 17.6 | 39.7 |
| Estriol (E3) | Boston | 3.9 | 14.2 | 3.8 | 13.7 |
| nmol $l^{-1}$ | Shanghai | 6.3 | 22.0 | 4.8 | 19.3 |
| Prolactin | Boston | 50.4 | 95.9 | 35.0 | 83.5 |
| µg $l^{-1}$ | Shanghai | 63.3 | 116.4 | 50.6 | 103.4 |
| Progesterone | Boston | 135.3 | 269.2 | 129.2 | 253.4 |
| nmol $l^{-1}$ | Shanghai | 143.4 | 248.3 | 138.2 | 231.6 |
| Growth hormone | Boston | 3.1 | 1.0 | 2.8 | 0.8 |
| mU $l^{-1}$ | Shanghai | 3.7 | 1.8 | 1.5 | 1.2 |
| Albumin | Boston | 40.5 | 36.7 | 39.7 | 36.1 |
| g $l^{-1}$ | Shanghai | 43.1 | 39.6 | 43.2 | 38.8 |
| SHBG[a] | Boston | 372.1 | 436.1 | 345.9 | 408.1 |
| nmol $l^{-1}$ | Shanghai | 429.7 | 478.2 | 414.6 | 518.6 |

[a]Sex hormone-binding globulin.

was no effect when our results were adjusted for maternal age and parity.

*L Lipworth[1,2] and Chung-cheng Hsieh [3,4]*
*[1]International Epidemiology Institute, Rockville, MD; [2]Vanderbilt-Ingram Cancer Center, Vanderbilt University School of Medicine, Nashville, TN; [3]Cancer Center, University of Massachusetts Medical Center, Worcester, MA; [4]Department of Epidemiology and Harvard Center for Cancer Prevention, Harvard School of Public Health, Boston, MA, USA*

doi: 10.1054/ bjoc.2000.1449, available online at http://www.idealibrary.com on IDEAL®

### Serum lactate dehydrogenase isoenzyme 1 as a prognostic predictor for metastatic testicular germ cell tumours

**Sir**

In a recent letter to the editor, Shamash et al (2000) argued that a raised serum lactate dehydrogenase catalytic concentration (S-LD) prior to induction chemotherapy for patients with germ cell tumours predicted a poor outcome. They referred to increasing evidence for a raised S-LD before first-line chemotherapy being as good if not better than serum human chorionic gonadotropin concentration (S-hCG), as found in, e.g., International Germ Cell Cancer Collaboratory Group (IGCCCG) (von Eyben et al, 1983; Mead and Stenning, 1997). Correspondingly, the fifth edition of the TNM (T =

 *© 2000 Cancer Research Campaign*

primary tumour, N = lymph node, M = distant metastases) classification included S-LD, serum alpha fetoprotein concentration, and S-hCG as serum tumour markers for testicular germ cell tumours (TGCT) (Sobin and Wittekind, 1997). The TNM classification is well documented and has been validated repeatedly.

LD consists of five LD isoenzymes. The literature points to a characteristic S-LD isoenzyme pattern in patients with TGCT and a raised S-LD: they predominantly have a raised S-LD isoenzyme 1 catalytic concentration (S-LD-1) (von Eyben et al, 1983). In contrast, patients with a raised S-LD due predominantly to S-LD isoenzyme 5 catalytic concentration most likely have presence of another disease, not TGCT. The S-LD-1 pattern in patients with TGCT reflects a characteristic chromosomal abnormality in the tumours: a high copy number of the short arms of chromosome 12, 12p, often with an isochromosome of 12p, i(l2p) (von Eyben et al, 1992a).

We combined the results of two published series of patients with metastatic TGCT monitored with S-LD-1 in Table 1 (von Eyben et al, 1992b; 2000). Even though the patients were treated at two institutions using different staging systems and treatments, the two series showed consistent findings. 42 of 81 patients (52%) had a raised S-LD-1 and 41 of the 42 also had a raised S-LD. However, eight patients had a discordant pattern with a normal S-LD-1 and a raised S-LD (Figure 1). Separated in three subgroups according to the S-LD-1 level, the 81 patients differed markedly regarding the survival ($P < 0.00001$, log-rank test, chi square for trend) (Figure 2A). Overall S-LD, S-hCG, and the prognostic classification of the IGCCCG study also predicted the survival ($P$ = 0.00032, 0.02, and 0.0005, respectively, Figure 2B and 2C) whereas S-AFP did not ($P = 0.68$) (Figure 2D). The subgroup of 39 patients with a normal S-LD-1 had the best survival and this favourable survival was not influenced whether S-LD was normal (31 patients) or raised (eight patients) ($P = 0.56$) (Figure 2E). Eight patients with an S-LD-1/S-LD fraction > 0.375 had a poorer survival than the 73 with a lower fraction ($P = 0.002$) (Figure 2F). So S-LD-1 might add prognostic information even for the TGCT patients with a raised S-LD. In one of the series, S-LD isoenzyme 5 catalytic concentration did not have a prognostic significance

**Table 1** Subgroups of the patients with metastatic TGCT according to S-LD-1 and the serum S category of the serum marker levels in the fifth edition of the TNM classification

| Serum tumour marker | S categories | Number of patients (*n* (%)) |
|---|---|---|
| S-LD-1 | < 12 U l$^{-1}$ | 39 (48%) |
| | 112–1120 U l$^{-1}$ | 37 (46%) |
| | > 1120 U l$^{-1}$ | 5 (6%) |
| S-LD | < 675 U l$^{-1}$ | 53 (65%) |
| | 675–4500 U l$^{-1}$ | 22 (29%) |
| | > 4500 U l$^{-1}$ | 5 (6%) |
| S-AFP | < 999 µg l$^{-1}$ | 76 (94%) |
| | 1000–10 000 µg l$^{-1}$ | 4 (5%) |
| | > 10 000 µg l$^{-1}$ | 1 (1%) |
| S-hCG | < 4999 IU l$^{-1}$ | 72 (91%) |
| | 5000–50,000 IU l$^{-1}$ | 4 (5%) |
| | > 50 000 IU l$^{-1}$ | 5 (6%) |
| IGCCCG | Good prognosis | 49 (60%) |
| | Intermediate prognosis | 16 (20%) |
| | Poor prognosis | 16 (20%) |

Serum categories cover normal, raised and very raised S-LD-1 and the tumour marker prognostic categories of the fifth edition of the TNM classification for S-LD, S-AFP and S-hCG. The TNM classification did not include S-LD-1.

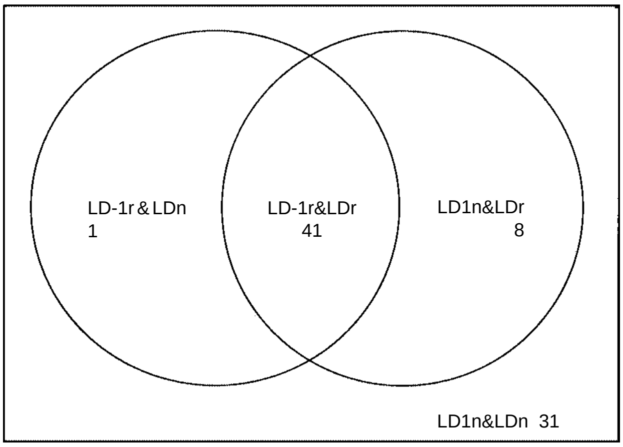

**Figure 1** Venn diagram shows the association between raised levels of S-LD and S-LD-1 for 81 patients with metastatic TGCT. LD1r&LDr denotes 41 patients with a raised S-LD-1 and S-LD and LD1n&LDn denotes 31 patients with a normal S-LD-1 and S-LD

(von Eyben et al, 2000). Accordingly, the prognostic implication of a raised S-LD might be solely due to a raised S-LD-1.

S-LD-1 gave a larger difference in survival between the subgroups of patients with the best and the poorest survival than the IGCCCG classification. So the TNM classification for patients with TGCT may not be the optimum usage of the available prognostic information.

These observations indicate that S-LD-1 is better than S-LD to predict the survival of patients with TGCT. However, the findings should be validated in larger series of patients with TGCT. We welcome interested oncologists to participate in a multicentre study of this question.

*FE von Eyben[1], EL Madsen[2], F Liu[3], R Amato[4] and H Fritsche[3]*
*[1]Medical Research Unit, Amtsrådhuset, Torvet, PO Box 142, Ringkoebing, Denmark; [2]Department of Oncology, Soenderborg Hospital, Denmark; [3]Division of Clinical Chemistry and [4]Department of Genitourinary Medical Oncology, Texas University MD Andersen Cancer Center, Honston, USA*

### REFERENCES

Mead GM and Stenning S for the International Germ Cell Cancer Collaboratory Group (1997) International germ cell consensus classification. A prognostic factor-based staging system for metastatic germ cell cancers. *J Clin Oncol* **15**: 594–603

Sobin LH and Wittekind C (1997) *TNM classification of malignant tumours* , 5th edn. Wiley-Liss: New York

Shamash J, Oliver RTD, Gallagher CJ, Newlands AC, Lister TA, Kelsey S, Gupta RK and O'Doherty CA (2000) Pre-induction LDH as a prognostic factor for outcome of high dose chemotherapy (HDCT) for germ cell tumours relapsing or refractory to conventional chemotherapy. *Br J Cancer* **82**: 2022–2023

von Eyben FE, Skude G, Fosså SD, Klepp O and Børmer O (1983) Serum lactate dehydrogenase (S-LDH) and S-LDH isoenzymes in patients with testicular germ cell tumors. *Mol Gen Genet* 189: 326–333

von Eyben FE, de Graaff W, Marrrink J, Blaabjerg O, Sleijfer DJ, Schraffordt Koops H, Oosterhuis JW, Petersen PH, van Echten-Arends J and de Jong B (1992*a*) Serum lactate dehydrogenase isoenzyme 1 activity in patients with testicular germ cell tumors correlates with the total number of copies of the short arms of chromosome 12 in the tumor. *Mol Gen Genet* **235**: 140–146

von Eyben FE, Blaabjerg O, Madsen El, Petersen PH, Smith-Sivertsen C and Gullberg B (1992*b*) Serum lactate dehydrogenase isoenzyme 1 and tumour volume are indicators of response to treatment and predictors of prognosis in metastatic testicular germ cell tumours. *Eur J Cancer* **238**: 410–415

von Eyben FE, Liu FJ, Amato RJ, Fritsche HA (2000) Lactate dehydrogenase (LD) isoenzyme 1 is the most important LD isoenzyme in patients with testicular germ cell tumor. *Acta Oncol* **39**: 509–517

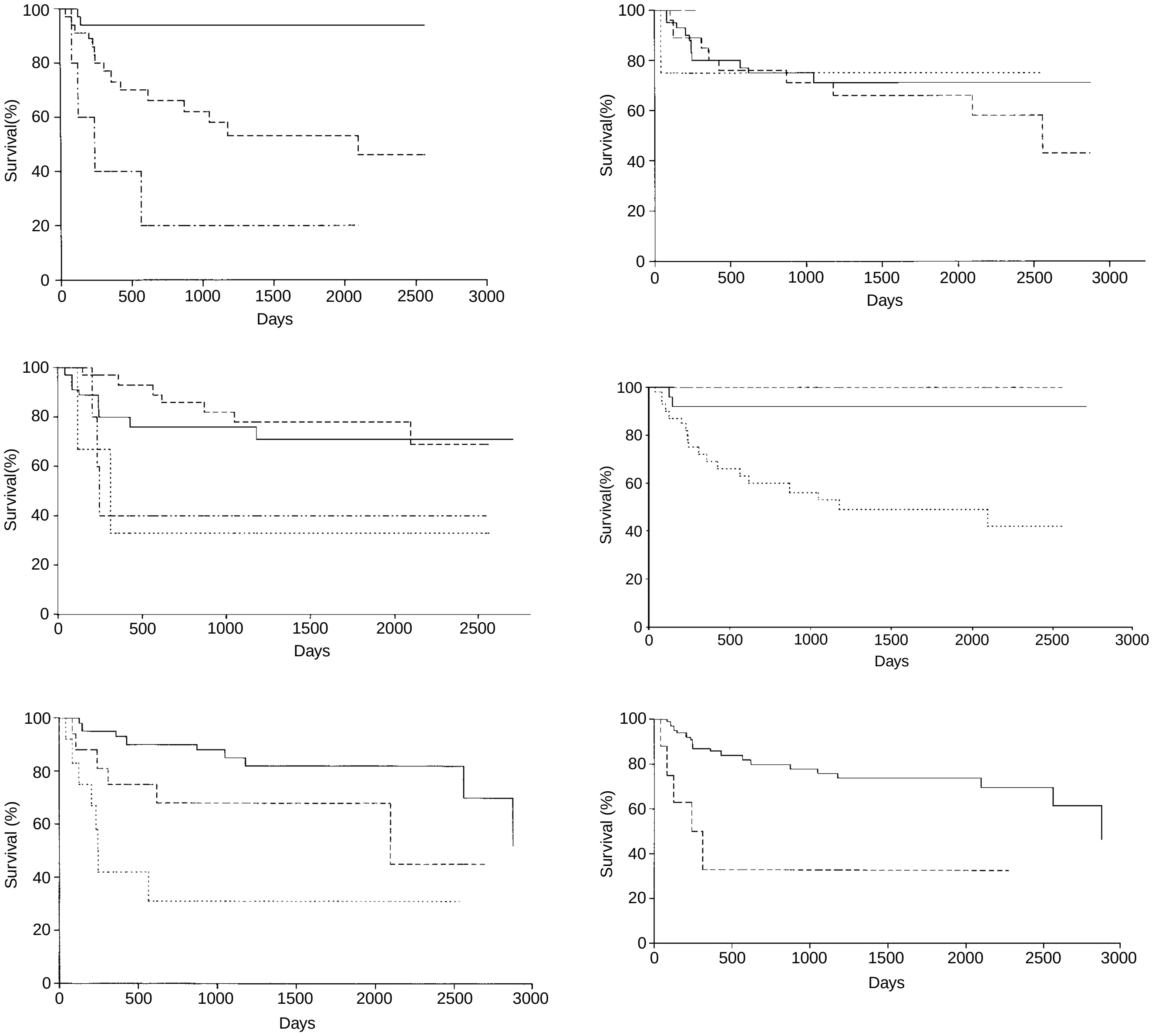

**Figure 2** (**A**) The survival for 81 patients with metastatic TGCT according to the initial postsurgical S-LD-1 (—— = 39 patients with S-LD-1 < 112 U L$^{-1}$, – – = 37 with S-LD-1 112–1120 U l$^{-1}$, and – · – = five with S-LD-1 > 1120 U l$^{-1}$). (**B**) The survival according to S-hCG (—— = 39 patients with S-hCG < 30 IU l$^{-1}$, – – = 33 with S-hCG 30–4999 IU l$^{-1}$, - - - = four with S-hCG 5000–50 000 IU l$^{-1}$, - - - = five with S-hCG > 50 000 IU l$^{-1}$). (**C**) The survival according to the risk groups of the IGCCCG classification (—— = 49 patients with a good prognosis, —— = 16 with an intermediate prognosis, and - - - = 16 with a poor prognosis). (**D**) The survival according to S-AFP (—— = 50 patients with S-AFP < 24 μg l$^{-1}$, —— = 26 with S-AFP 24–999 μg l$^{-1}$, - - - = four with S-AFP 1000–10 000 μg l$^{-1}$, - - - = one with S-AFP > 10 000 μg l$^{-1}$. (**E**) The survival according to the concomitantly normal and raised levels of S-LD-1 and S-LD (—— = 31 patients with normal values of S-LD-1 and S-LD, — — = eight with a normal value of S-LD-1 and a raised S-LD, and - - - = 42 with a raised S-LD-1 (with or without a raised S-LD)). (**F**) The survival according to the S-LD-1/S-LD ratio (—— = 73 patients with an S-LD-1 ratio < 0.375, and – – = eight with a ratio > 0.375)

doi: 10.1054/ bjoc.2000.1450, available online at http://www.idealibrary.com on IDEAL®

### Serum lactate dehydrogenase isoenzyme 1 as a prognostic predictor for metastatic testicular germ cell tumours – reply

**Sir**

von Eyben et al describe their experience of splitting total LDH into its various isoenzymes and have identified LDH isoenzyme 1 (S-LD-1) as having prognostic significance in untreated patients with metastatic germ cell tumours. The evidence they present is clearly impressive and S-LD-1 appears to be able to identify patients with a poor outcome as effectively as the IGCCCG specification or serum HCG alone.

 *© 2000 Cancer Research Campaign*

